# Mutation detection in cholestatic patients using microarray resequencing of *ATP8B1* and *ABCB11*

**DOI:** 10.12688/f1000research.2-32.v2

**Published:** 2013-03-20

**Authors:** Kirsten E McKay, Christopher K Bruce, Jane L Hartley, A S Knisely, Ulrich Baumann, Sonja-Stephanie Bockisch, Ekkehard Sturm, Christian J Hendriksz, Deidre A Kelly, Fiona Macdonald, Paul Gissen

**Affiliations:** 1 University of Birmingham, Edgbaston, Birmingham, B15 2TT, UK; 2 Birmingham Women’s Hospital, Birmingham, B15 3TG, UK; 3 Birmingham Children’s Hospital, Birmingham, B4 6NH, UK; 4 Institute of Liver Studies, King's College Hospital, London, London, SE5 9RS, UK; 5 Medizinische Hochschule Hannover, Hannover, 130625, Germany; 6 University of Tübingen, Tübingen, 72074, Germany; 7 Salford Royal NHS Foundation Trust, Salford, M6 8HD, UK; 8 Great Ormond Street Hospital for Children, London, WC1N 3JH, UK

## Abstract

**Background** **: **Neonatal cholestasis is a common presentation of childhood liver diseases and can be a feature of various conditions including disorders of bile acid biogenesis and transport, various inborn errors of metabolism and perinatal infections. Some inherited metabolic diseases can be easily screened using biochemical assays, however many can only be accurately diagnosed by DNA sequencing. Fluorescent capillary Sanger sequencing (FS) is the gold standard method used by clinical laboratories for genetic diagnosis of many inherited conditions; however, it does have limitations. Recently microarray resequencing (MR) has been introduced into research and clinical practice as an alternative method for genetic diagnosis of heterogeneous conditions. In this report we compared the accuracy of mutation detection for MR with FS in a group of patients with ‘low-normal’ gamma glutamyl transpeptidase (gGT) cholestasis without known molecular diagnoses.

**Methods** **:** 29 patient DNA samples were tested for mutations in the *ATP8B1* and *ABCB11* genes using both FS and MR. Other known causes of “low gGT cholestasis” such as ARC syndrome and bile acid biosynthesis disorders were excluded.

**Results** **:** Mutations were identified in 13/29 samples. In 3/29 samples FS and MR gave discordant results: MR had a false positive rate of 3.4% and a false negative rate of 7%.

**Conclusions** **:** The major advantage of MR over FS is that multiple genes can be screened in one experiment, allowing rapid and cost-effective diagnoses.  However, we have demonstrated that MR technology is limited in sensitivity. We therefore recommend that MR be used as an initial evaluation, with FS deployed when genetic and clinical or histopathological findings are discordant.

## Introduction

Neonatal cholestasis is characterised by persistent hyperbilirubinaemia and has an incidence of around 1 in 2500 live births ^1^. It can occur as a result of impaired bile acid biosynthesis, defective bile secretion by hepatocytes (due to membrane transporter defects), or extrahepatic obstruction to biliary flow. Patients present with jaundice, dark urine, pale acholic stools and hepatomegaly. Older infants may have pruritus, develop fat-soluble vitamin deficiencies and fail to thrive ^1^. The aetiology of cholestasis is varied and includes multiple inherited causes, such as progressive familial intrahepatic cholestasis (PFIC), Niemann-Pick disease type C and arthrogryposis, renal dysfunction and cholestasis (ARC) syndrome ^1^. Furthermore cholestasis can be subdivided into ‘low-normal’ and high gamma glutamyl transpeptidase (gGT) categories depending on the level of serum gGT activity.

Accurate diagnosis is essential in order to inform decisions on treatment and management. As an example, familial intrahepatic cholestasis 1 (FIC1) protein deficiency caused by mutations in *ATP8B1* (PFIC type 1) and bile salt export protein (BSEP) deficiency caused by mutations in *ABCB11* (PFIC type 2) have similar presentations, however liver transplantation is used more frequently in PFIC type 2 then type 1 ^6^. Genetic testing allows accurate assessment of the genetic risk of cholestasis in families, in addition to providing definitive diagnoses. Clinical laboratories tend to use fluorescent capillary sequencing (FS; Sanger method), because it lends itself to automation and is highly sensitive. However, FS is limited by machine capacity and relatively high consumables costs.

Microarray resequencing (MR) is an alternative sequencing method previously used to detect mutations in patients with intrahepatic cholestasis ^2^. We have performed initial testing of a 300kb custom-designed microarray (Birmingham ReseqUencing Microarray, BRUM1) in patients with known mutations ^4^. BRUM1 includes probes to sequence two genes ( *ATP8B1* and *ABCB11)* involved in PFIC. This short research article describes further evaluation of the BRUM1 microarray using samples simultaneously tested by FS and MR in patients without previously known mutations and assesses the advantages and disadvantages of MR in clinical laboratory use.

## Materials and methods

### DNA samples

DNA samples from 29 children (15 females) who presented with intrahepatic cholestasis in the first year of life, low-normal range gGT activity without ARC syndrome or defects in bile acid biosynthesis were used. ARC syndrome was excluded by clinical examination ^3^ and bile acid biosynthesis disorders were excluded by determination of urinary bile acid profile by electrospray ionisation mass spectrometry. Thus there was a high likelihood of detecting mutations in *ATP8B1* and *ABCB11* in these samples.

### Polymerase chain reaction

Polymerase chain reaction was used to amplify the coding exons of *ATP8B1* and *ABCB11* plus the intron-exon boundaries prior to both FS and MR. The PCR reaction used 50ng of DNA, 0.5μM each primer (Sigma), 0.5mM dNTPs (Bioline) and 2 units of Taq DNA polymerase (Biomix Red, Bioline UK) in 1x PCR buffer.

### Fluorescent sanger sequencing

5μl of PCR product was treated with ExoSap IT (GE Healthcare) for 30 mins at 37°C before being denatured at 85°C for 15 mins. Sequencing primers (at a final concentration of 0.5μM) was added to 1μl of BigDye 3.1 and 1x BigDye buffer (Applied Biosystems) in a final volume of 10μl. The sequencing programme was thermal cycling for 34 cycles of denaturing at 95°C for 20 seconds, annealing 50°C for 20 seconds and extension at 60°C for 4 minutes. Resulting products were precipitated using EDTA/Na acetate and ethanol and centrifuged at 4000 rpm for 30 minutes. Products were washed in 70% ethanol before being air dryed, resuspended in 10μl of HiDi formamide (Applied Biosystems) and denatured. Resuspended products were analysed using a DNA analyzer 3730xl (Applied Biosystems) and the resulting sequence traces were analysed using Sequence Analysis 5.2.2 (Applied Biosystems).

### Microarray resequencing

PCR product quantitation, pooling, fragmentation, labelling and hybridisation and was performed according to the GeneChip Custom Resequencing Array Protocol V2.1 (Affymetrix). Arrays were washed and stained using a FS450 fluidics station before being scanned with a GCS3000 7G scanner (Affymetrix). Intensity files were generated using AGCC (Command Console V1.0, Affymetrix) and processed in GSeq 4.1 (Affymetrix). Base calling assumed the diploid model and a quality score threshold of 2 (default settings were used for all other parameters).

## Results

*ATP8B1* and *ABCB11* were analysed using FS and MR. The sequence variants detected in 13 of the samples (44.5% detection rate) are listed in Table 1. Eight variants had previously been described in association with PFIC and ten variants were novel. Four of the variants were nonsense mutations (22%), two were splice site changes (11%) and the rest were missense (67%). No insertions or deletions were detected using the specifically designed probes. No mutations were detected by either technique in the remainder of the patients and therefore the cause of ‘low-normal gGT’ cholestasis remains undetermined in these cases. The negative results in these cases may be explained by whole exon deletions or duplications, intronic mutations affecting splicing, or promoter region mutations in *ATP8B1* and *ABCB11*, as such mutations would not be detected by either strategy. In addition, it is possible that further genes are involved in the phenotype of neonatal cholestasis with low gGT.

**Table 1.** The sequence variants identified in this study.

Sample	Gene	DNA change ^b^	Protein change ^c^	Zygosity	Predicted effect	Previously reported?	Microarray resequencing	Fluorescent sanger sequencing
2	*ABCB11*	c.850G>C	p.V284L	Heterozygous	Missense	Yes ^7^	Y	Y
4	*ABCB11*	c.2178+1G>T		Heterozygous	Altered splicing	Yes ^8^	Y	Y
6	*ATP8B1*	c.1010T>G	p.M337R	Heterozygous	Missense	Novel ^d^	N	Y
	*ATP8B1*	c.1018A>G	p.M340V	Heterozygous	Missense	Novel ^d^	N	Y
7	*ATP8B1*	c.208G>A	p.D70N	Heterozygous	Missense	Yes ^9^	Y	Y
9	*ABCB11*	c.2170G>A	p.D724N	Heterozygous	Missense	Novel ^d^	Y	Y
12	*ABCB11*	c.2611-2A>T		Heterozygous	Altered splicing	Yes ^10^	Y	Y
17	*ATP8B1*	c.1660G>A	p.D554N	Homozygous	Missense	Yes ^11^	Y	Y
	*ATP8B1*	c.1564G>A ^a^	p.D522N ^a^	Heterozygous ^a^	Missense	Novel ^d^	Y	N ^a^
18	*ABCB11*	c.290T>G	p.L97*	Homozygous	Truncated protein	Novel	Y	Y
19	*ATP8B1*	c.3040C>T	p.R1014*	Heterozygous	Missense	Yes ^9^	Y	Y
22	*ATP8B1*	c.1014C>G	p.N338K	Heterozygous	Missense	Novel ^d^	N	Y
	*ATP8B1*	c.1018A>G	p.M340V	Heterozygous	Missense	Novel ^d^	N	Y
	*ABCB11*	c.1636C>A	p.Q546K	Heterozygous	Missense	Novel ^d^	Y	Y
25	*ABCB11*	c.499G>A	p.A167T	Heterozygous	Missense	Yes ^12^	Y	Y
	*ABCB11*	c.3458G>A	p.R1153H	Heterozygous	Missense	Yes ^10^	Y	Y
26	*ABCB11*	c.3484G>T	p.E1162*	Homozygous	Truncated protein	Novel	Y	Y
29	*ABCB11*	c.483C>A	p.C161*	Homozygous	Truncated protein	Novel	Y	Y

^a^ - Variant was not confirmed by FS and is therefore a false positive finding.

^b^ - DNA changes were experimentally determined by sequencing.

^c^ - Protein changes are predicted rather than experimentally determined.

^d^ - Novel missense changes are of unclear pathogenicity.

In sample 17, MR detected a variant not confirmed by FS, constituting a false positive result. Two samples (6 and 22) had compound heterozygous changes in *ATP8B1* detected by FS but not by MR. Both samples had two variants in close proximity; therefore we speculate that these are in *cis* and that each sequence variant has impaired the hybridisation of surrounding probes. Our experience of testing patients for mutations in these genes suggests that false negative results arising in this manner are likely to be uncommon. However, in this cohort they occurred in 7% of samples.

## Discussion

MR is an attractive alternative to traditional FS for genetic testing in neonatal cholestasis. The BRUM1 microarray used in this study has a much larger capacity than was utilised in this experiment, as it contains probes for 92 genes associated with inherited disorders. Analysis of the ability of this microarray to detect known gene mutations has shown a 97% mutation detection rate for base substitutions ^4^. The major advantage of MR is increased capacity, allowing sequencing of multiple genes in one experiment as quickly as one gene using FS. The main bottleneck is the requirement to quantify and to pool individual PCR products before hybridisation. This step is time-consuming and prone to error (due to pipetting volume variations and potential sample mix-ups). Therefore, automation to minimise such errors would be useful, if not essential, for adoption of this method into clinical laboratories. Alternatively, use of long range PCR as the preparatory step for the MR protocol could reduce the number of reactions to be pooled, although it is more sensitive to poor DNA quality than standard PCR. Finally, microfluidics-based PCR systems, such as the 48.48 Access Array (Fluidigm Corporation, San Francisco, CA), might be combined with MR to avoid the quantification and pooling step altogether. This system allows the simultaneous but separate amplification of up to 48 PCR products for up to 48 samples, in nanolitre-sized reactions, using a semi-automated process. The end product of the process is a pool of PCR products for each sample, and it is commonly used for target enrichment for next generation sequencing experiments.

Of the mutations recorded in the Human Gene Mutation Database ^5^ in *ATP8B1* and *ABCB11*, 24% and 16% were small insertions or deletions (indels) respectively, though their combined frequency in PFIC cases is unknown as most are private mutations. The major disadvantage of MR is it’s insensitivity for the detection of indels ^4^. Whilst known indels can be detected with proper microarray design, novel indels will be missed. Larger insertions and deletions involving whole exons, of the type routinely detected by multiplex ligation-dependent probe amplification (MLPA; MRC-Holland, Netherlands), are not detected by either MR or FS. Another disadvantage, underscored by the results of this study, is that mis-called base substitutions (both false negative and false positive calls), are frequent and were found in approximately 10% of samples in this study.

In summary, MR allows rapid and cost-effective genetic screening in neonatal cholestasis and yields results relevant for patient management. Many clinical laboratories are experienced in oligoarray comparative genome hybridization (CGH) and thus have access to equipment required for MR, suggesting that MR could be implemented for a relatively small monetary investment. In principle, MR could be applied to many clinical scenarios involving heterogeneous conditions, especially if the mutations tend to be base substitutions. We recommend that MR methods be used for initial evaluation, with FS deployed when genetic and clinical or histopathologic findings are discordant.

In recent years, various next generation sequencing (NGS) platforms have become available which allow massively parallel resequencing experiments to be performed. In particular ‘benchtop’ sequencers like the GS Junior (Roche), Personal Genome Machine (Life technologies) and MiSeq (Illumina) are attractive to clinical laboratories because they allow fast, cheap resequencing of multiple genes, and when the capacity is used optimally the costs are lower than MR. This study has identified a significant rate of discordant results obtained by MR when compared to Sanger sequencing, and consequently we predict that NGS will be the method of choice for clinical laboratory resequencing tests.
