# Supplementary material for: Mutation detection in cholestatic patients using microarray resequencing of ATP8B1 and ABCB11
Source: F1000Res. 2013 Mar 20;2:32. Originally published 2013 Feb 6. [Version 2] doi: 10.12688/f1000research.2-32.v2 (PMC3907151; doi:10.12688/f1000research.2-32.v2)
Supplement: Peer review history

# Peer review: Referee response for version 2

**Published:** 2013-09-13

**Authors:** Kornelia Neveling

**Affiliations:** 1 Radboud University Nijmegen Medical Centre, Nijmegen, Netherlands

This is a well-written article. The authors already discuss the weaknesses of their technique, therefore I do not need to comment on this further. However, I would have liked to see some more information on the identified variants. There is no information given about the predicted pathogenicity of the identified (missense) variants. This would be necessary in order to draw any conclusion on whether the disease-causing mutations have been identified or not.

I have read this submission. I believe that I have an appropriate level of expertise to confirm that it is of an acceptable scientific standard.

# Peer review: Referee response for version 2

**Published:** 2013-08-29

**Authors:** Struan Grant

**Affiliations:** 1 Center for Applied Genomics, Children's Hospital of Philadelphia Research Institute, University of Pennsylvania, Philadelphia, PA, USA

The authors have adequately addressed all outstanding concerns.

I have read this submission. I believe that I have an appropriate level of expertise to confirm that it is of an acceptable scientific standard.

# Peer review: Referee response for version 1

**Published:** 2013-02-14

**Authors:** Andrea Ballabio

**Affiliations:** 1 Telethon Institute of Genetics and Medicine, Naples, Italy

The authors compare, in a very thorough and detailed manner, the performance of Microarray resequencing (MS) vs. Sanger sequencing (FS) for mutation detection in the *ATP8B1* and *ABCB11* genes in cholestatic patients. They recommend the cautious use of microarray resequencing as an initial evaluation tool and propose to limit the use of Sanger sequencing to more problematic and discordant cases.

However, given the non-negligible discordance observed by the authors between the two approaches (MR had a false positive rate of 3.4% and a false negative rate of 7%), the definition of the cases that should be analyzed by FS is not entirely clear. Moreover, a number of other resequencing procedures that can be applied to the simultaneous analysis of multiple genes are now available. Some of them are very competitive in terms of efficacy and cost with MS and it is quite likely that in the near future they will be more widely used with respect to MS. Ideally, the authors should have discussed about this issue.

I have read this submission. I believe that I have an appropriate level of expertise to confirm that it is of an acceptable scientific standard, however I have significant reservations, as outlined above.

# Peer review: Referee response for version 1

**Published:** 2013-02-14

**Authors:** Struan Grant

**Affiliations:** 1 Center for Applied Genomics, Children's Hospital of Philadelphia Research Institute, University of Pennsylvania, Philadelphia, PA, USA

I have read this submission. I believe that I have an appropriate level of expertise to confirm that it is of an acceptable scientific standard.
